# Molecular Mechanisms of Probiotic Action Against Gastrointestinal Cancers

**DOI:** 10.3390/ijms26167857

**Published:** 2025-08-14

**Authors:** Christina Thoda, Maria Touraki

**Affiliations:** Laboratory of General Microbiology, Department of Genetics, Development and Molecular Biology, School of Biology, Faculty of Sciences, Aristotle University of Thessaloniki (A.U.TH.), 54 124 Thessaloniki, Greece; christhoda@bio.auth.gr

**Keywords:** probiotics, gut microbiota, gastrointestinal cancers, postbiotics, encapsulation, next-generation probiotics

## Abstract

Gastrointestinal (GI) cancers represent a major global health burden. Among them, colorectal cancer (CRC) is the most common type, followed by esophagus, stomach, liver, and pancreatic cancer. Since disturbance of the gut microbiota has been directly associated with the development of severe health issues, including cancer, probiotic administration may induce dysbiosis reversion and ameliorate carcinogenesis. Therefore, manipulation of the gut microbiota composition based on probiotic utilization has gradually attained scientific interest as a potent therapeutic modality for GI cancers. This review aims to synthesize the current in vitro and in vivo evidence on probiotics’ effectiveness in GI cancer chemoprevention and treatment. It also provides a classification of the fundamental anticancer features of probiotics, including antiproliferation and cell death induction, anticarcinogenic compound production, reduction in chemotherapy-related toxicity, gut microbiota modulation, intestinal barrier improvement, antioxidant activity, immunomodulatory/anti-inflammatory effects, and carcinogen detoxification. Finally, it underscores the future perspectives and challenges of probiotic administration to individuals. In this regard, it emphasizes the exploitation of advanced encapsulation techniques and the development of novel genetically engineered probiotics and next-generation probiotics as feasible ways to improve their bioavailability, ensure their targeted delivery, and eliminate their mild side effects to the host’s health.

## 1. Introduction

Gastrointestinal (GI) cancers account for a significant majority of cancer incidence and mortality worldwide, thus representing a major global health burden. Among them, colorectal cancer (CRC) is the most common type of gastrointestinal malignancy, followed by esophageal, stomach, liver, and pancreatic cancer. Understanding the composite molecular mechanisms behind the expeditious development of these types of cancer is of utmost importance, especially for the exploration of novel treatment modalities [1,2,3,4]. Accumulating evidence underlines the intricate bidirectional interactions between the gut microbiota and the host’s cells, as well as the implication of commensal microbes imbalance, known as dysbiosis [5], in the occurrence of specific types of GI cancers [6,7,8]. The gut microbiota, which includes bacteria, viruses, and yeasts, is a diverse and dynamic ecosystem comprising trillions of microorganisms. It is predominantly composed of two bacterial phyla, Bacillota and Bacteroidota, which represent more than 90% of the total microbial community. Other subdominant phyla include Pseudomonadota, Actinomycetota, Verrucomicrobiota, and Fusobacteriota. A healthy gut microbiota serves as an “external metabolic organ” for the host, with profound impact on nutrient and energy metabolism, intestinal epithelial barrier protection, and production of bioactive metabolites [9,10,11]. Additionally, the gut microbiota can interact with the host immune system to safeguard the host’s health against invading pathogens via immunomodulation. Targeting the gut microbiota [12,13,14,15,16,17] as well as the intratumoral microbiota [18,19,20,21] to ameliorate carcinogenesis may serve as promising and evolutionary approaches in precision-medicine anticancer treatments.

The gut microbiota can shape oncologic outcomes via multiple pathways. For instance, due to its vast metabolic capacity, it can affect the pharmacokinetics of common chemotherapeutics, thus contributing to their therapeutic efficacy and safety [22,23,24,25,26]. Furthermore, it is responsible for the regulation of many biological processes, including oxidative stress, inflammation, and immunity [27,28,29,30,31]. Given the growing evidence that indicate the strong correlation between the gut microbiota and cancer development, designing microbiota-oriented strategies for CRC [32,33,34,35,36,37,38,39,40,41,42,43,44,45,46], gastric [47,48,49], liver [50,51,52,53] and pancreatic [54,55,56,57,58] cancer prevention and treatment comprises a challenging task in the field of oncology and molecular medicine. In parallel, microbiota-derived metabolites, such as bioactive peptides and short-chain fatty acids (SCFAs), can either suppress tumor growth directly or synergistically with chemotherapy or immunotherapy [59,60,61,62,63,64,65].

Bacteriotherapy employs certain bacteria, or their products, to selectively target cancer cells or activate the immune system to combat cancer. In recent years, among the bacteria that can be harnessed for therapeutic purposes, probiotics have gained scientific interest due to their beneficial and multifaceted effects on human health [66,67]. Probiotics represent a non-pathogenic distinct group of microorganisms that contribute to the maintenance of gut microbiota homeostasis. It is widely accepted that probiotics are involved in different intestinal functions, including epithelial immune modulation, digestion, nutrient absorption, metabolism, and angiogenesis. They also play a vital role in the amelioration of digestive disorders (e.g., lactose intolerance, irritable bowel syndrome, ulcerative colitis, and others) [68,69,70,71,72,73,74,75,76]. Several studies have demonstrated probiotics’ potential to serve as an effective anticancer treatment [77,78,79,80,81,82].

This review provides a comprehensive description of current knowledge on the anticancer properties of probiotics. Furthermore, it emphasizes the underlying molecular mechanisms that configure probiotics’ effects on GI malignant cells. Finally, it demonstrates the recent findings regarding the advantages of probiotic application as a main or complementary approach for the management of GI cancers, as well as the development of effective techniques to overcome the challenges derived from their administration to weakened individuals.

## 2. Mechanisms of Probiotic Action Against Gastrointestinal Cancers

**Figure 1:**
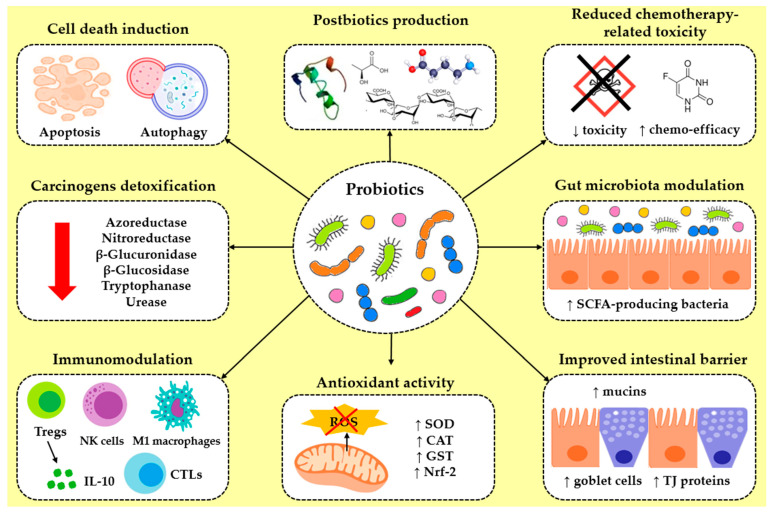
Diagrammatic representation of the main mechanisms by which probiotics suppress GI cancers: (1) cell death induction (apoptosis or autophagy), (2) postbiotic production, (3) reduced chemotherapy-related toxicity (reduction indicated by a black cross) and enhanced chemotherapeutic efficacy, (4) gut microbiota modulation via dominance of SCFA-producing bacteria, (5) improved intestinal barrier function through increased mucin secretion from goblet cells, (6) antioxidant properties via diminishing ROS accumulation (elimination is indicated by a red cross) and induction of antioxidant enzymes’ activity, (7) immunomodulatory and anti-inflammatory effects, and (8) carcinogen detoxification.

Probiotics, which are defined as the viable, non-pathogenic microorganisms, are distinguished for their health-promoting properties to the host. Conventional probiotics are frequently found in traditional fermented foods and primarily comprise bacterial strains from the genera *Lactobacillus*, *Bifidobacterium*, *Lactococcus*, *Pediococcus*, *Streptococcus*, *Bacillus*, and *Enterococcus*, as well as the yeast *Saccharomyces* [83]. The European Food Safety Authority (EFSA), the World Health Organization (WHO), and the Food and Agriculture Organization (FAO) have established a list of mandatory guidelines to ensure probiotic safety in terms of clinical use. Several prerequisites are considered during the screening and selection process of the appropriate probiotic strains for human consumption, including strain identification, resistance to antibiotics, stability through the gastrointestinal tract (GIT), acid and bile tolerance, and adhesion ability on the intestinal epithelial cells (IECs) [84]. Certain probiotics exhibit a profound potential to suppress GI cancers [85,86,87] based on the following mechanisms: (1) antiproliferation and cell death induction [88,89,90], (2) anticarcinogenic compound production [91], (3) reduction in chemotherapy-related toxicity [92,93], (4) gut microbiota modulation [94], (5) intestinal barrier improvement [95], (6) antioxidant activity [96], (7) immunomodulatory [97,98] and anti-inflammatory [99,100,101] effects, and (8) carcinogen detoxification [102,103] (Figure 1).

### 2.1. Antiproliferation and Cell Death Induction

The antiproliferative and pro-apoptotic properties of probiotics have been reported on various GI cancers, including CRC [83,104,105,106,107], gastric [108], liver [109], and pancreatic cancer [110]. Several in vitro and in vivo studies have revealed the potential benefits of probiotic administration in apoptosis induction, which serves as the primary target in cancer treatment advancement. Probiotics, mainly originated from the genera *Lactobacillus* [111,112,113,114,115,116,117,118,119,120,121,122,123,124,125], *Bifidobacterium* [126,127,128,129,130], *Bacillus* [131], *Lactococcus* [132], *Clostridium* [133,134,135], *Enterococcus* [136], *Streptococcus* [137], *Saccharomyces* [138,139,140], as well as mixed probiotic formulations [141,142,143,144], have the ability to suppress the uncontrolled cellular proliferation of cancer cells, predominantly via the activation of apoptosis. They can trigger the intrinsic apoptotic pathway as indicated by mitochondrial membrane potential (MMP) loss [121,122,126,145] with subsequent release of cytochrome C [121,145]. Additionally, increased caspase expression [117,119,121,123,126,130,140,144,146], up-regulation of the pro-apoptotic Bax protein, and down-regulation of the anti-apoptotic Bcl-2 protein have been observed [116,118,119,121,123,126,143,144,146]. Recently, two distinct studies have revealed that probiotics could elicit immunogenic cell death (ICD), a type of regulatory programmed cell death associated with the release of damage-associated molecular patterns (DAMPs), which function as signaling molecules promoting adaptive immune responses [125,146].

The link between diverse signaling pathways and apoptosis induction via probiotic administration is well established. For instance, probiotics have been found to inhibit the nuclear factor-kappa B (NF-κB) [120,122,134,147,148,149], phosphatidylinositol 3-kinase (PI3K)/AKT [122,150], and Wnt/β-catenin pathways [133,151] in malignant cells, thus eliminating cancer progression. Moreover, probiotic-induced alterations in the expression levels of cell cycle-related genes are also correlated with apoptosis. An in vitro study demonstrated that the *L. paracasei* subsp. *paracasei X12* treatment upregulates p27, a cell cycle cyclin-dependent kinase inhibitor, suppresses the mammalian target of rapamycin (mTOR)/4EBP1 signaling pathway, induces G1 phase arrest in HT-29 cells, and suppresses cyclin E1 expression [152]. In another study, a mixed formulation of *Lactobacillus* spp. significantly downregulated cyclin A, cyclin B1, cyclin B2, and cyclin E in Caco-2 cells [153]. Probiotics can also upregulate tumor-suppressor genes, such as p53 [118,130,154] and Phosphatase and Tensin Homolog (PTEN) [150,155], as well as downregulate proto-oncogene K-ras expression [118,155], leading to apoptotic cell death of cancer cells. Furthermore, probiotics can inhibit the epidermal growth factor receptor (EGFR) signaling pathway to eradicate tumor development [138,142], while they can also trigger autophagy to mitigate inflammation and counteract GI malignancy [145,156,157,158,159,160,161,162].

Angiogenesis is a critical process in cancer development and metastasis, since the formation of new blood vessels permits tumor cells to enter the bloodstream and disseminate to secondary distant locations. Probiotics have been shown to inhibit the production of proangiogenic factors, such as the vascular endothelial growth factor (VEGF) [163,164,165,166], while they can also reduce vasculogenic mimicry (VM) [164]. In this context, *Saccharomyces boulardii* attenuated intestinal inflammation and promotes mucosal tissue recovery in vivo, via inhibition of VEGF-induced angiogenesis [163]. Finally, probiotics may hinder the epithelial–mesenchymal transition (EMT) progression, a state where epithelial cells lose their ability to adhere to other cells and become more invasive, by preventing the expression of transcription factors. *Clostridium butyricum* inhibited EMT and VM formation of CRC cells through methyltransferase-like 3 (METTL3) downregulation [164].

### 2.2. Anticarcinogenic Compounds Production

According to the International Scientific Association of Probiotics and Prebiotics (ISAPP) definition, postbiotics are defined as the “inanimate microorganisms and/or their components that are beneficial to the host [167]”. Postbiotics encompass a heterogeneous group of bioactive molecules including heat-killed bacteria, bacterial lysates, cell wall fragments, cell-free supernatants (CFSs), exopolysaccharides (EPSs), extracellular vesicles (EVs), SCFAs, organic acids, bacteriocins, enzymes, neurotransmitters, vitamins, and amino acids [168,169,170,171]. Postbiotics can modulate multiple core signaling pathways associated with cellular proliferation and growth, as well as they can selectively induce apoptosis in cancer cells without affecting healthy cells [172,173,174,175,176,177]. Furthermore, they play a fundamental role in mediating communication between the resident microbiota and the immune system, thus governing intestinal homeostasis via local and systemic immune responses modulation [167,178,179].

Numerous in vitro and in vivo studies emphasized the anticancer properties of various postbiotics. CFSs obtained from *Lactobacillus* [180], *Bifidobacterium* [181], *Bacillus* [182], *Enterococcus* [183], *Pediococcus* [184], and *Saccharomyces* [185] are implicated in apoptosis activation in cancer cells. Additionally, CFS from the probiotic *Aspergillus oryzae* containing heptelidic acid decreased the proliferation of pancreatic cancer cells through the p38 mitogen-activated protein kinase (MAPK) signaling pathway [186], while postbiotic metabolites from *Weizmannia coagulans* MZY531 suppressed colorectal tumorigenesis in CT26 colorectal tumor-bearing mice by regulating apoptosis and autophagy [187]. Postbiotics also exert anti-inflammatory and antioxidant activities, thus effectively preventing dextran sodium sulfate (DSS)-induced colitis in vivo [188,189,190].

EPSs exert their tumor-suppressive effects via activation of the extrinsic Fas/FasL-mediated apoptotic pathway [191,192], induction of autophagy [193,194,195], and suppression of the NF-κB signaling pathway, as indicated in a recent in vivo study [196]. Probiotic-derived EVs are multifaceted entities composed of numerous bioactive metabolites that collectively contribute to their anticancer properties [197,198,199]. For instance, *L. buchneri* EVs exhibited apoptotic activity by increasing pro-apoptotic genes expression in HT-29 and AGS cell lines [200], while *L. paracasei* EVs inhibited CRC cells’ growth by decreasing HIF-1α-mediated glycolysis [201]. Among SCFAs, butyrate has been found to display epigenetic effects, such as alterations of DNA methylation and selective histone acetylation, thus promoting cell-cycle arrest and apoptosis [202]. Several studies have reported that conjugated linoleic acid (CLA) exerts its antiproliferative and apoptotic effects by increasing peroxisome proliferator-activated receptor gamma (PPAR-γ), which regulates lipid metabolism [149,203,204]. Additionally, CLA might have an impact on cell cycle control, thus diminishing differentiation, survival, and growth via p53-dependent or -independent pathways [205,206]. In parallel, *Lactobacillus plantarum*-derived indole-3-lactic acid (ILA) ameliorated colorectal tumorigenesis via enhancement of CD8+ T cells’ function through epigenetic mechanisms [207].

Dietary folate participates in the regulation of several key molecular procedures, including DNA damage repair, apoptosis, and cell cycle control. Several probiotic strains, especially those belonging to *Lactobacillus* and *Bifidobacterium* species, enhance folate biosynthesis in the colon, which is crucial for maintaining genomic stability, thus leading to CRC prevention [208]. An in vitro study demonstrated that two distinct strains of *Streptococcus thermophilus*, namely M17PTZA496 and TH982, exhibit potent anticancer activity and folate production [209]. Additionally, the administration of the folate-producing strain *Streptococcus thermophilus* CRL 808 attenuated the 5-fluorouracil (5-FU)-induced intestinal mucositis and contributed to the effectiveness of 5-FU in a mouse model [210].

Gamma-aminobutyric acid (GABA) not only acts as an inhibitory neurotransmitter in the central nervous system (CNS), but also modulates several GIT functions since it is recognized as a chemical messenger by two main types of G-protein-coupled receptors found within the enteric nervous system (ENS): the ionotropic GABA_A_ receptor (GABA_A_R) and the metabotropic GABA_B_ receptor (GABA_B_R) [211]. A recent study found that GABA-producing *L. plantarum* exhibits anti-proliferative and anti-migration effects against 5-FU-resistant HT-29 cells, which are mediated by the GABA_B_R signaling pathway activation. This pathway is associated with apoptosis induction via inhibition of cAMP-dependent ERK-CREB phosphorylation and cellular inhibitor of apoptosis protein 2 (cIAP2) expression [212].

### 2.3. Reduction in Chemotherapy-Related Toxicity

Numerous anticancer medications, including 5-FU, oxaliplatin, doxorubicin, cisplatin, celecoxib, tamoxifen, irinotecan, and gemcitabine, have the ability to interact synergistically with probiotics. Probiotics in conjunction with anticancer drugs enhance apoptosis and antitumor efficacy, minimize side effects, control drug resistance, and suppress recurrence [213,214,215,216,217]. Prophylactic treatment with *L. rhamnosus* GG in combination with celecoxib reduced the tumor burden in a CRC animal model via upregulation of the pro-apoptotic Bax protein and downregulation of the anti-apoptotic Bcl-2 protein [118]. Furthermore, co-administration of probiotics with celecoxib reduced COX-2 expression [118,218,219]. Probiotics [220,221] and postbiotics [222,223] were found to improve the antitumor and apoptotic efficacy of 5-FU in a dose-dependent manner, while they can also mitigate the severity of diarrhea symptoms and intestinal mucositis after FOLFOX treatment [224]. Another study showed that the combination of *L. plantarum*-derived postbiotics with 5-FU increased the sensitivity of 5-FU-resistant CRC cells [225]. In parallel, *B. infantis* ameliorated chemotherapy-induced mucositis in a CRC rat model after 5-FU treatment through decreased levels of pro-inflammatory cytokines, while it reduced Th1 and Th17 response and enhanced CD4+ CD25+ Foxp3+ Tregs response [226]. Studies also suggest that *Bifidobacterium* downregulates the NF-κB-dependent genes, thus leading to cancer prevention [227]. On the other hand, probiotics enhance chemotherapeutic effectiveness via oxidative stress induction to trigger cell death. Probiotic-derived bioactive metabolites from *Lactobacillus* spp., including SCFAs and EPSs, reduced the viability of both sensitive and chemo-resistant HT-29 cells through mitochondrial reactive oxygen species (ROS) production and enhanced doxorubicin-induced toxicity [228].

### 2.4. Gut Microbiota Modulation

One of the fundamental mechanisms through which probiotics can prevent cancer development is the modulation of gut microbiota. Among pathogenic bacteria, *Helicobacter pylori*, *Clostridium difficile*, *Fusobacterium nucleatum*, *Escherichia coli*, and *Porphyromonas gingivalis* have been repeatedly associated with an elevated risk for GI cancer occurrence. Probiotics can selectively inhibit these carcinogenic pathogens employing several antimicrobial mechanisms, such as competitive exclusion, secretion of antimicrobial peptides (AMPs), and immune system modulation [229]. Due to the existence of specific structural elements exposed to their outer cell envelope (e.g., EPSs, lipoteichoic acids, and surface layer proteins), probiotics are responsible for adhesion onto IECs, which in turn hinder pathogens colonization in the gut [230]. Probiotics can also lower the intestinal pH through increased secretion of SCFAs, thus inhibiting the growth of harmful bacteria and maintaining intestinal homeostasis.

Probiotics play a fundamental role in altering gut microbiota diversity and abundance [129,137,139,146,159,162,166,224,231,232,233,234,235,236,237,238,239,240], leading to accelerated recovery of its composition and function [241] and dysbiosis reversion [120,130,144,242,243,244], in animal models with induced GI malignancy. Commonly, the dominance of SCFA-producing bacteria at the expense of pathogenic bacteria and bile acid-biotransforming bacteria in the gut [133,134,151,243,245] has been shown to be drastically effective in tumorigenesis amelioration. For instance, *Limosilactobacillus fermentum* GR-3 intervention reversed the expansion of *Dubosiella*, *Pseudoflavonifractor*, and *Bacteroides* induced by azoxymethane (AOM)/DSS in mice model and maintained a higher abundance of SCFA-producing bacteria, including *Alloprevotella*, *Lachnospiraceae*_NK4A136_group, *Rikenella*, *Bifidobacterium*, and *Muribaculum intestinale* [239]. In another study, probiotics were found to suppress hepatocellular carcinoma growth in mice by causing a shift in the gut microbiota towards beneficial bacteria, such as *Prevotella* and *Oscillibacter*, known for their ability to produce anti-inflammatory metabolites [234]. Administration of *L. salivarius* Ren also increased the amount of *Prevotella* in the 1,2-dimethylhydrazine (DMH)-treated group [232]. Additionally, the gut microbiota is implicated in metabolome alterations, potentially influencing cancer progression. Increased levels of beneficial metabolites, such as SCFAs, indole-3-carboxylic acid (ICA), indole-3-propionic acid (IPA), vitamin B12, and vitamin D3, and decreased levels of harmful secondary bile acids are indicative of carcinogenesis suppression [239,246]. A lower bioavailability of some amino acids (glutamic acid, aspartic acid, threonine, and serine), which serve as potent energy sources for proliferation and biosynthetic pathways, may also portray a metabolic disadvantage for cancer cells [245]. In mice with AOM/DSS-induced cancer, administration of *Bifidobacterium bifidum* CGMCC 15068 led to tumor incidence reduction through alterations in metabolites involved in glycolysis, the tricarboxylic acid (TCA) cycle, and fatty acid biosynthesis [247]. Moreover, ILA derived from *L. gallinarum* was shown to promote apoptosis and inhibit tumorigenesis in vivo [237].

### 2.5. Intestinal Barrier Improvement

The intestinal barrier serves as the primary line of defense against harmful pathogens. It facilitates nutrient absorption and contributes to the maintenance of intestinal homeostasis. Disruption of its integrity can trigger gut leakage, inflammation, and an extensive immunological response. In cancer patients, impairment of intestinal barrier function and increased intestinal permeability are often observed. Probiotic intake can activate the regeneration of the gut barrier by promoting mucin production by goblet cells. The administration of *L. coryniformis* MXJ32 significantly suppressed the total tumor number in mice with AOM/DSS-induced CRC and restored the population of goblet cells [243]. Mucins are known to eliminate the possibility of pathogens and carcinogenic compounds coming into contact with IECs [149,248,249]. An in vivo study has proved the protective effect of the probiotic mixture VSL#3 against Muc2 mucin-deficient mice by dampening colitis symptoms. These effects were mainly attributed to the acceleration of tissue regeneration, reduction in pro-inflammatory cytokines, ROS depletion, restoration of the gut microbiota abundance, and increased secretion of AMPs and SCFAs in the tumor microenvironment (TME) [250]. Other probiotics fortify the intestinal mucus layer via autophagy [251,252] and calcium-dependent signaling pathways [252]. Furthermore, strengthening tight junctions (TJs) enables protection against intestinal inflammation. Probiotic gavage reduced the intestinal leakage and increased the expression levels of TJ proteins, including occludin, claudin-1, and ZO-1 [149,241,243,253,254]. Finally, postbiotics derived from *Bacillus* [255] and *Lactobacillus* spp. [256] were found to enhance the gut barrier integrity.

### 2.6. Antioxidant Activity

Probiotics display antioxidant activity through various mechanisms, including free radical scavenging, synthesis of antioxidant metabolites (e.g., glutathione or vitamins like C and E), gut microbiota modulation, autophagy [257], and induction of antioxidant ROS-scavenging enzymes expression [154,258] to eliminate the cumulative production of ROS in cells with redox imbalance [259]. Additionally, probiotics can also suppress metastasis and angiogenesis, reduce oxidative stress by inhibiting cytokine production, and modulate signaling pathways, such as nuclear factor erythroid 2-related factor 2 (Nrf-2), NF-κB, and MAPK, thus allowing them to exert their beneficial antioxidant effects on cancer patients. This antioxidant potential has been observed across various probiotic strains, especially those belonging to the *Lactobacillus* and *Bifidobacterium* genera [260]. Interestingly, oral administration of *L. plantarum* AS1 in rats with DMH-induced CRC suppressed lipid peroxidation and upregulated the expression of the antioxidant enzymes superoxide dismutase (SOD), glutathione S-transferase (GST), and catalase (CAT), leading to tumorigenesis eradication [261]. In another study, intake of *Lactococcus lactis*, a CAT-producing bacterium, prevented DMH-induced CRC in mice as evidenced by increased CAT activity, which led to significantly lower levels of H_2_O_2_, limited inflammatory colonic damage, and tumor shrinkage in comparison with the untreated animals [262]. Substantial research suggests that probiotics can activate the Nrf-2 signaling pathway in host cells in order to inhibit oxidative stress and inflammation. The Nrf-2 transcription factor represents a pivotal element that disconnects from its constant inhibitor, Keap1, when ROS levels are elevated. Then, the Nrf-2 migrates to the nucleus, where it forms a complex with the antioxidant response element (ARE) sequences, thereby initiating the transcription of genes correlated with antioxidation. Several in vitro studies have illuminated the role of Nrf-2 pathway activation upon probiotic administration. Pretreatment of enterocytes with *L. casei* Shirota prevented the loss of membrane integrity and eliminated 2,2′-Azobis (2-Amidinopropane) Dihydrochloride (AAPH)-induced oxidative stress via modulation of the Nrf2/Keap-1 signaling [263]. Additionally, *Bacillus coagulans* T242 exerted antioxidant effects by diminishing ROS levels and activating the Nrf2 signaling pathway in HT-29 cells [264].

### 2.7. Immunomodulatory and Anti-Inflammatory Effects

Probiotics are capable of interacting with the IECs in the GIT, thus inducing immunomodulation and suppressing inflammatory responses through the restoration of intestinal microbial balance [97]. Through the enhancement of the host’s immunity, probiotics facilitate the identification and elimination of tumor cells [158]. Their immunomodulatory properties vary between individuals and are mostly attributed to the release of cytokines and chemokines from immune cells. Probiotics have been found to display anti-inflammatory properties, which are related to the downregulation of Toll-like receptor (TLR) expression [265,266] and the subsequent inhibition of NF-κΒ signaling pathway in IECs [85,267]. Apart from the NF-κB pathway, probiotics are also involved in the regulation of the JAK/STAT, the MAPK, and the PI3K/AKT/mTOR signaling pathways via the secretion of cytokines and AMPs, thus promoting the mucosal and systemic immune response [268]. The expression of *JAK* and *STAT* genes following probiotic treatment with *Lactobacillus* spp. and *Bifidobacterium* spp. (either independently or in combination) was evaluated in HT-29 cells. Additionally, pro-inflammatory genes participating in the NF-κB pathway (including *TIRAP*, *IRAK4*, *RIP*, and *NEMO*) and production of pro-inflammatory cytokines (IL-6 and IL-1β) were investigated. The probiotic cocktail downregulated the expression of *JAK* genes and the inflammatory genes of the NF-κB pathway, while the production of IL-6 and IL-1β was decreased. Thus, researchers have indicated the significance of probiotics as a potential protective treatment in inflammatory bowel disease (IBD) [269]. Upon adherence to the IECs, probiotics induce the production of cytokines, leading to the activation of the key mediators in maintaining gut homeostasis, Tregs [270]. Probiotics were found to elevate the production of anti-inflammatory cytokines (e.g., IL-10, TGF-β) to the detriment of pro-inflammatory cytokines in the affected colonic mucosa, a mechanism that may reduce malignancy progression [142,144,244,271]. Moreover, probiotic administration has been related to local recruitment of cytotoxic T lymphocytes (CTLs) [272,273,274] and natural killer (NK) cells in the TME [275] associated with increased cytotoxicity. *L. rhamnosus* Probio-M9 intervention also strengthened anti-PD-1-based immunotherapy response by promoting the dominance of beneficial gut bacteria and increased production of butyric acid, α-ketoglutaric acid, N-acetyl-L-glutamic acid, and pyridoxine, therefore improving immunotherapeutic response [273].

The exploitation of probiotics might be of great assistance in combating the pro-inflammatory state that is associated with cancer. It is well known that one of the main causes of cancer development is chronic inflammation, which can either exacerbate tumor progression or elicit acute inflammatory reactions. Under chronic inflammation conditions, increased expression levels of pro-inflammatory cytokines can promote activation of oncogenes [276,277]. Probiotics exert their anti-inflammatory capacity via different pathways [278,279,280]. For instance, they mitigate colonic inflammation in vivo [134,159], both mediated by the histamine H2 receptor [281] or with concurrent reductions in IL-6, IL-1β, and increased IL-10 concentrations [282]. Probiotics are capable of significantly downregulating the expression levels of IL-8 in LPS- [283], flagellin- [147], or *Salmonella typhimurium*-treated cancer cells [284]. Interestingly, probiotic administration in mice models led to suppression of pro-inflammatory cytokines secretion, notably iNOS and IL-6, thus contributing to limited intestinal and liver tissue inflammation [285]. Nonetheless, probiotics are also known to directly promote macrophage polarization, potentially shifting them from the M2 towards the M1 phenotype, which is associated with enhanced phagocytic activity and anti-tumor immune response [97]. Finally, decreased NLRP3-mediated colitis and inflammation-associated CRC were observed after *E. faecalis* KH2 gavage in CRC mice [286].

### 2.8. Carcinogen Detoxification

Probiotics contribute to cancer prevention by degrading or deactivating carcinogenic substances, thus enabling the neutralization of their adverse effects on the gut microbiota [287]. Enzymes like azoreductase and nitroreductase can metabolize xenobiotics to produce dangerous aromatic amines [288]. Regular probiotic intake can minimize the number of harmful bacteria in the gut with a subsequent reduction in the synthesis of carcinogenic substances [205]. Several probiotics, including *Lactobacillus* and *Bifidobacterium* strains, are known to lack the enzyme 7α-dehydroxylase, which is responsible for converting primary bile acids into secondary bile acids. This deficiency is crucial, and it means that probiotics are less likely to contribute to the generation of harmful secondary bile acids, a trait that is associated with the increased probability of developing CRC. Recent studies have also shown the ability of probiotics to impede the function of bacterial enzymes that biotransform exogenous substances into more potent carcinogens, such as bacterial nitroreductases [289], β-glucuronidases [127,238,289,290], β-glucosidases [127,290], tryptophanases, and ureases [127]. Collectively, probiotics may reduce the risk of cancer occurrence via boosting the ability of the host to metabolize and remove carcinogens.

In conclusion, probiotics have emerged as promising anticancer agents against different types of GI cancers, as indicated by in vitro (Table 1) and in vivo studies (Table 2).

## 3. Challenges and Future Perspectives

Although probiotics present multiple benefits, mild side effects have been previously observed, including bloating, gas, diarrhea, constipation, nausea, headaches, migraines, and skin reactions. For most people, these symptoms usually improve gradually within a few weeks. However, probiotic supplementation should be employed with caution in weakened populations, such as immunocompromised or transplant patients, people with a history of infective endocarditis, and people with extensive intestinal damage [291,292,293]. Furthermore, another disadvantage is the survival rate of probiotics, which is usually reduced after oral administration in vivo. For that reason, novel encapsulation biomaterials and advanced strategies, such as microfluidics and bioprinting, have been developed to improve probiotic bioavailability in the host. Alginate/chitosan or starch-based nanoparticles, as well as liposomes with encapsulated probiotics, effectively shield them from the harsh gastrointestinal conditions and enable their targeted delivery [294,295,296,297]. In recent years, numerous studies have demonstrated that encapsulated probiotics are effective in various in vitro and in vivo applications. For instance, *Bacillus amyloliquefaciens*-loaded nanoparticles attenuated colonic inflammation, oxidative stress, and apoptosis in a DSS-induced colitis model [298]. Oral delivery of encapsulated *L. plantarum* CRD7 modified the gut microbiota composition, enhanced the intestinal barrier function, and augmented antioxidant and immunomodulatory effects in vivo [299]. An in vitro study showed that *L. acidophilus*-derived silver nanoparticles exhibit antiproliferative effects and induce apoptosis in hepatocellular carcinoma HepG2 cells through suppressing the AMPK/mTOR signalling pathway [300]. Oral drug delivery of encapsulated probiotics has also been implicated in CRC treatment [301]. Microencapsulated *L. plantarum* LAB12 was found to be associated with apoptosis induction and anti-angiogenesis to confer chemoprevention in CRC mice [302]. Moreover, oral administration of prebiotics-encapsulated probiotic spores could specifically increase the overall richness of the gut microbiota, elevate the abundance of SCFA-producing bacteria, and ameliorate CRC in mice [303]. However, there are still impediments prior to clinical use of these formulations, since researchers should also take into consideration how to improve the stability and the adhesion properties of coated probiotics on the IECs [294,304].

Developing new types of probiotics suitable for targeted delivery is still mandatory to overcome the complex GIT conditions. Recent studies emphasize expanding the range of candidate probiotics via the utilization of multiple gene editing tools to prepare genetically engineered probiotics. Most of these strategies focus on *Escherichia coli* Nissle 1917 (EcN) due to its known genome. Some genetic engineering approaches have also been performed on *Lactobacillus* spp., *Bifidobacterium* spp., and *Lactococcus* spp. Thus, implementing robust genetic manipulation strategies for other probiotics are highly desired for obtaining novel functional probiotics for enhanced intestinal delivery [305,306,307,308,309,310,311]. Several in vivo and in vitro studies have employed engineered probiotics to determine whether they display enhanced anticancer efficacy against various GI malignancies. Interestingly, an orally administered, yeast-based therapeutic with the ability to secrete “miniature” immune checkpoint inhibitors significantly reduced intestinal tumor burden [312]. In another study, a chitosan/sodium alginate-coated probiotic EcN was genetically engineered to overexpress CAT and SOD. This formulation improved the intestinal barrier function, improved the abundance of *Lachnospiraceae*_NK4A136 and *Odoribacter* in the gut microbiota, and eliminated intestinal inflammation in an IBD mouse model [313]. Intake of the engineered probiotic EcN, which synthesizes the ketone body 3-hydroxybutyrate, promoted the growth of probiotic bacteria, especially *Akkermansia* spp., and ameliorated DSS-induced colitis in mice [314]. Furthermore, the probiotic EcN that was genetically engineered to secrete IL-2 regulated the innate immune responses and the gut microbiota, thereby leading to relief from inflammation in DSS-induced IBD [315]. Nonetheless, it is still necessary to develop suitable preservation and large-scale production techniques to manage the widespread application of the engineered probiotics. It is definitely indispensable to evaluate the biosafety and in vivo fate of the engineered probiotics individually or in synergy with other anticancer agents. Despite these challenges, it is expected that engineered probiotics will play a crucial role in future anticancer treatments [316,317].

A new emerging trend is the utilization of next-generation probiotics (NGPs). NGPs are distinct probiotics that differ from the conventional ones and have been identified using large-scale genomic testing as probiotic strains with potential health benefits. They mainly belong to the genera *Bacteroides*, *Akkermasia*, *Faecalibacterium*, and *Eubacterium*. These new candidates represent a very promising solution in cancer elimination [318,319,320,321,322,323,324,325,326,327,328,329]. For example, *Akkermansia muciniphila* intervention improved tumor cell accumulation in CRC mice via modulating the NF-κB pathway, cell apoptosis, and the gut microbiota composition [330]. In another study, *Roseburia intestinalis* protected against CRC by producing butyrate, while it also improved anti-PD-1 efficacy by inducing functional CD8^+^ T cells [331]. Although it is still early, accumulating evidence indicates that NGPs can provide targeted and customized treatments for GI cancers [320].

Additional preclinical data and well-designed randomized clinical trials are still required to fully comprehend the relationships between cancer and probiotics. Probiotics have been evaluated as adjuvant therapies with promising anticancer effects in recent clinical trials. Particularly, strains of *Lactobacillus* and *Bifidobacteria* were reported to lower the mortality risk and reduce the chemotherapy-related adverse effects in CRC patients. Most in vivo and in vitro experiments focus on determining the health-promoting properties of a single probiotic strain. On the other hand, probiotic formulations commonly used in clinical trials contain a great variety of probiotics, providing possible advantages via synergistic interactions. However, inadequate design of studies often leads to contradictory results and misconceptions. It is also important to underline that certain probiotic strains induce unique responses in each individual, while many commercially available probiotics lack strict standards for safety [217]. Collectively, based on the aforementioned knowledge, outlining large-scale clinical trials is necessary to identify potential beneficial probiotics for cancer prevention and treatment.

## Figures and Tables

**Table 1:** In vitro effects of probiotics on gastrointestinal cancer cell lines.

Cancer Type	Probiotic	Cell Line	Effect/Mode of Action	Reference
**Colorectal**	*Bacillus coagulans* T242	HT-29	↑ Nrf-2/Keap1 pathway-related protein expression, ↑ antioxidant enzymes (GSH, CAT, SOD), ↓ MDA, ↓ pro-inflammatory cytokines (IL-6, IL-8, TNF-α)	[264]
	*Bacillus polyfermenticus* KU3	LoVo, HT-29	↓ proliferation	[131]
	***Bifidobacterium*** **spp.**			
	*B*. *adolescentis* SPM0212	HT-29, SW480, Caco-2	↓ proliferation, ↓ TNF-α, changes in cellular morphology	[127]
	*B. longum*	LoVo, SW480, SW1463	↓ proliferation, ↓ migration	[129]
	*B. longum* D42	HT-29	↓ proliferation, ↑ LDH release, ↑ apoptosis, ↑ ROS, ↑ caspase-3, -9, ↑ Bax/↓ Bcl-2, ↓ MMP	[126]
	*Clostridium butyricum*	HCT-116	168 DEPs enriched in apoptosis and inflammatory pathways, ↓ NFKB1 protein level	[135]
	*C*. *butyricum* ATCC 19398	HCT-116, Caco-2	↓ METTL3 expression, ↓ vimentin, ↓ *VEGFR2*, ↓ EMT, ↓ VM formation	[164]
	*Enterococcus faecium* FP51	Caco-2	↓ proliferation, ↑ adherence to cancer cells, ↑ SCFAs bioproduction	[246]
	*E. faecium* RM11	Caco-2	↓ proliferation	[136]
	*Escherichia coli* Nissle 1917	CT26	↑ Bax/Bcl-2 ratio, ↑ caspase-3, synergistic enhancement of Gal anti-tumor efficacy by ICD induction	[146]
			↑ apoptosis, ↓ MMP, PINK1/Parkin pathway activation, ↑ mitophagy, MPTP disruption, cytochrome C release	[145]
	***Lactobacillus*** **spp.**			
	*L*. *acidophilus* CICC 6074	HT-29	↓ proliferation in a dose-and time-dependent manner, ↑ apoptosis, ↓ MMP, cytochrome C release, ↑ caspase-3, -9, ↑ Bax/↓ Bcl-2	[121]
	*L. acidophilus KLDS1.0901*	HT-29, Caco-2	↓ proliferation in a dose-dependent manner, ↑ apoptosis, ↓ MMP, ↓ NF-κB and PI3K/AKT pathways	[122]
	*L. casei* ATCC 393	HT-29	↓ proliferation in a dose-and time-dependent manner, ↑ apoptosis	[115]
		CT26, HT-29	↓ proliferation, ↑ caspase-3, ↑ ICD	[125]
	*L*. *casei* Shirota	Caco-2/TC7	prevention of membrane barrier disruption, ↓ ROS accumulation, ↑ GPX2 expression, ↓ p65 phosphorylation, ↑ Nrf-2 pathway	[263]
	*L. fermentum* RM28	Caco-2	↓ proliferation	[136]
	*L. fructosus* C2	Caco-2	↓ dextran permeability, ↓ IL-8, ↓ p-ERK and p-JNK after cells infection with ETEC or *Salmonella typhimurium*	[284]
	*L*. *kefiri* SGL 13	HT-29	↓ proliferation correlated with the eIF2 and RXR activation pathways, ↑ Bax, ↓ IL-8 in cells stimulated with LPS	[283]
	*L. paracasei*, *L. brevis*	HT-29	↓ proliferation, ↑ apoptosis, ↑ Bax, ↑ caspase-3, -9, ↓ Bcl-2	[119]
	*L. paracasei* IMPC2.1	DLD-1	↓ proliferation, ↑ apoptosis	[113]
	*L. paracasei* subp. *paracasei X12*	HT-29	G1-phase arrest, ↓ cyclin E1, ↑ p27, ↓ mTOR/4EBP_1_ pathway	[152]
	*L. rhamnosus* (Probio-M9)	Caco-2	prevention of LPS-induced damage of tight junction integrity	[241]
	*L. rhamnosus* GG	Caco-2	↓ IL-8 in cells stimulated by flagellin, ↓ NF-κB pathway	[147]
		HT-29, HCT-116	apoptotic-related nuclear morphological changes, ↑ caspase-3, ↑ Bax, ↓ Bcl-2, ↓ cyclin D1, mitochondrial function impairment	[123]
	*L*. *salivarius* FP25	Caco-2	↓ proliferation, ↑ adherence to cancer cells, ↑ SCFAs bioproduction	[246]
	*L*. *salivarius* FP35			
	*Lactococcus lactis* NK34	DLD-1, HT-29, LoVo	↓ proliferation	[132]
	*Ligilactobacillus salivarius* LZZAY01	CT26, HCT-116, SW620, NCM460	↓ proliferation, ↑ autophagy and apoptosis	[162]
	*Saccharomyces boulardii*	HT-29, SW480, HCT-116	↓ EGFR-Erk and EGFR-Akt pathways, ↑ apoptosis, ↓ HER-2, ↓ HER-3, ↓ IGF-1 receptor	[138]
	*S*. *cerevisiae*	SW480	↑ apoptosis, ↓ p-Akt1, ↓ Rel A, ↓ Bcl-XL, ↓ pro-caspase 3, -9, ↑ Bax, ↑ cleaved caspase-3, -9	[140]
	*Streptococcus thermophilus*	HCT-116, HT-29, Caco-2	↓ proliferation, cell cycle arrest, ↑ apoptosis	[137]
	*S. thermophilus* CRL 808	Caco-2	↑ cytotoxicity of 5-FU	[210]
	*S. thermophilus* M17PTZA496, *S. thermophilus* TH982	HT-29	anticancer activity via folate production	[209]
	*Pediococcus pentosaceus* FP3	Caco-2	↓ proliferation, ↑ adherence to cancer cells, ↑ SCFAs bioproduction	[246]
	Various *Lactobacillus* strains	HT-29	↓ proliferation, ↑ NO secretion, ↑ Bax/Bcl-2 ratio, ↑ LDH	[116]
	**Mixed formulations**			
	*Bifidobacteria* spp. cocktail	LS174T	↑ apoptosis, ↓ *EGFR*, ↓ *HER-2*, ↓ *PTGS-2*	[142]
	*Bifidobacterium bifidum* H3-R2 and *L. lactis* KLDS4.0325	HT-29	↑ caspase-3, -9, ↑ Bax, ↓ Bcl-2	[144]
	*Lactobacillus* spp. cocktail	HT-29	↓ proliferation, ↑ apoptosis, Notch and Wnt/β-catenin pathways modulation	[143]
	*L. acidophilus* ATCC 314 and *L. fermentum* NCIMB 5221	Caco-2, CRL-1831	↓ proliferation and ↑ apoptosis towards cancer cells, significant protection of normal colon cells	[141]
	*L. acidophilus* CL1285 and *L. casei* LBC80R	CRL-2134 (LS513)	improved dose-dependent apoptotic efficacy of 5-FU, ↑ caspase-3, ↓ p21	[220]
	*L. pentosus* B281 and *L. plantarum* B282	Caco-2	↓ proliferation, G1-phase arrest, ↓ *cyclins A*, *B1*, *B2*, *E*	[153]
	Probiotic cocktail	HT-29	↓ *JAK*, ↓ *TIRAP*, ↓ *IRAK4*, ↓ *NEMO*, ↓ *RIP*, ↓ IL-6, ↓ IL-1β	[269]
	Probiotic cocktail	HT-29	↑ autophagy genes (*PIK3C3*, *ATG14*, *Beclin*, *PIK3R4*, *ATG5*, *ATG16*, *ATG7*, and *ATG3*), anti-inflammatory effects	[160]
**Gastric**	*B. polyfermenticus* KU3	AGS	↓ proliferation	[131]
	*B. longum* subsp. *longum* 35624	AGS	↓ proliferation, ↓ COX-2 expression in combination with celecoxib	[219]
	*L. acidophilus* La-14 SD-5212			
	*L. paracasei* IMPC2.1	HGC-27	↓ proliferation, ↑ apoptosis	[113]
	*L. plantarum*	AGS, CRL-1739	↑ PTEN, ↓ AKT pathway	[150]
	*L. rhamnosus* GG	HGC-27	↓ proliferation, ↑ apoptosis, ↓ ODC activity	[111]
	*Lactococcus lactis* NK34	AGS	↓ proliferation	[132]
**Pancreatic**	*Aspergillus oryzae* ATCC 42149	SUIT2, Panc-1, MIA-PaCa-2	↓ proliferation via heptelidic acid production through the p38 MAPK pathway	[186]
	*L. casei* ATCC 39392 and *L. reuteri* ATCC 23272	PaCa-2, Panc-1, AsPC-1, BxPC-3	↓ proliferation, migration, and invasion via TLR4 suppression	[265]

↑: induction, increase or up-regulation, ↓: inhibition, decrease or down-regulation.

**Table 2:** In vivo effects of probiotics on animal models with induced malignancies.

Cancer Type	Probiotic	Animal Model	Effect/Mode of Action	Reference
**Colorectal**	***Bifidobacterium*** **spp.**			
	*B. adolescentis* SPM0212	male Sprague Dawley rats	↓ harmful fecal enzymes (β-glucuronidase, β-glucosidase, tryptophanase, and urease)	[127]
	*B. animalis* subsp. *lactis*BB12	male C57BL/6J mice with DSS- induced cancer	colitis amelioration, ↓ TNF-α, ↑ apoptosis	[128]
	*B. animalis* subsp. *lactis* SF	male pathogen-free BALB/c tumor- bearing mice	enhancement of irinotecan’s antitumor effect, ↓ tumor growth and invasion, ↓ intestinal inflammation, gut microbiota modulation, ↓ TGF-β leakage, ↓ PI3K/AKT pathway, ↑ autophagy, ↑ CD4^+^ and CD8^+^ T cells differentiation in tumor tissue	[159]
	*B. animalis* subsp. *lactis* TCI604	female C57BL/6 J mice with AOM/DSS- induced cancer	↓ colonic polyps, ↓ pro-inflammatory cytokines, ↓ inflammatory immune cells, ↓ NF-κB pathway, dysbiosis reversion	[244]
	*B. breve* CCFM683	male C57BL/6J Apc^Min+^ mice	↑ CLA levels, ↓ NF-κB pathway, ↑ MUC2, ↑ Claudin-1, ↑ ZO-1, ↑ tumor cell apoptosis via the CLA-PPAR-γ axis	[149]
	*B. infantis*	Sprague Dawley rats with DMH-induced cancer	↓ IL-6, ↓ IL-1β, ↓ TNF-α, ↓ Th1 and Th17 response, ↑ CD4^+^ CD25^+^ Foxp3^+^ Tregs response	[226]
		male BALB/c mice	↓ tumor growth, gut microbiota composition regulation, immune function enhancement	[129]
	*B. longum* SX-1326	C57BL/6 mice with AOM/DSS-induced cancer	↑ caspase-3, ↓ Bcl-2, ↑ p53 pathway, GBA regulation via restoration of damaged EC cells, ↓ release of 5-HT in brain tissue, dysbiosis reversion, ↓TLR4/MyD88/NF-κB pathway	[130]
	*Clostridium butyricum*	female Apc^min/+^ mice	↓ proliferation, ↑ apoptosis, ↓ Wnt/β-catenin pathway, ↓ pathogenic bacteria and bile acid-biotransforming bacteria, ↑ SCFA-producing bacteria, ↑ GPR43 and GPR109A	[133]
		male C57BL/6 mice with DSS-induced cancer	improved intestinal barrier function, ↑ TJ-related protein expression levels, ↓ TNF-α, ↓ IL-1β, ↓ IL-13, ↑ IL-10, ↓ oxidative stress, ↑ phosphorylation of Akt, mTOR and p70 ribosomal protein S6 kinase	[253]
		male C57BL/6J with AOM/DSS-induced cancer	↓ CRC incidence, ↓ inflammation, ↑ apoptotic cells in the tumor tissue, ↓ IL-6, ↑ IL-10, gut microbiota composition enrichment, ↓ MyD88 and NF-κB expression	[134]
	*C. butyricum* ATCC 19398	female BALB/c HCT-116 tumor- bearing mice	↓ tumor metastasis, ↓ EMT, ↓ VM formation	[164]
	*Enterococcus faecalis* KH2	C57BL/6 mice	↓ NLRP3-mediated colitis and inflammation-associated CRC	[286]
	*E. coli* Nissle 1917	CT26 tumor- bearing mice	antitumor effect via gut microbiota regulation, ↑ infiltration of CD8+ T cells into the ΤΜΕ	[146]
			↑ antitumor efficacy in synergy with the autophagy activator rapamycin	[145]
	***Lactobacillus*** **spp.**			
	*Lactobacillus* spp.	male Sprague Dawley rats with DMH- induced cancer	↓ angiogenesis/↓ inflammation after coadministration with telmisartan, ↑ programmed cell death, dysbiosis reversion, ↓ CEA levels	[165]
	*L. acidophilus*	male BALB/c mice with AOM-induced cancer	↓ colonic lesions, ↓ CEA and CA19-9 tumor markers, ↑ CD4^+^ and CD8^+^ cells number, ↑ IFN-γ and IL-10 serum levels	[271]
	*L. acidophilus* CGMCC 878	male Sprague Dawley rats with DMH- induced cancer	↓ tumor number, gut microbiota alteration, ↓ fecal β-glucuronidase	[238]
	*L. acidophilus* CICC 6074	female HT-29 tumor- bearing BALB/c mice	↑ apoptosis, cytochrome C release, ↑ Bax and Caspase-3, -9/↓ Bcl-2	[121]
	*L. acidophilus* KFRI342	male F344 rats with DMH-induced cancer	↓ aberrant crypt foci, ↓ *E. coli* number in fecal samples, ↓ β-glucuronidase and β-glucosidase activities	[290]
	*L. acidophilus* NCFM	female CT26 tumor-bearing BALB/cByJ mice	↓ tumor volume growth, ↓ colonic carcinogenesis, ↑ apoptosis, ↓ CXCR4 mRNA expression in the colon, ↓ MHC class I expression	[112]
	*L. acidophilus*, *B. bifidum*	male BALB/c mice with AOM-induced cancer	↓ miR-135b, miR-155, and *KRAS* expression, ↑ miR-26b, miR-18a, *APC*, *PU.1*, and *PTEN* expression	[155]
	*L. acidophilus*, *L. rhamnosus* GG	Sprague Dawley rats with DMH-induced cancer	↓ aberrant crypt foci, ↓ fecal nitroreductase activity, ↓ β-glucuronidase activity	[289]
			↓ aberrant crypt foci, ↓ β-catenin, ↓ NF-κB, ↓ COX-2 in conjuction with celecoxib	[218]
	*L. acidophilus*, *L. rhamnosus GG*	Sprague Dawley rats with DMH-induced cancer	↓ tumor multiplicity, ↑ Bax/↓ Bcl-2, ↓ K-ras/↑ p53 expression	[118]
	*L. casei* ATCC 393	female BALB/c mice	↓ tumor volume by 80%, ↑ TRAIL, ↓ survivin	[115]
		male Swiss mice with DMH-induced cancer	↓ CEA, ↓ aberrant crypt foci, ↑ p-JNK-1 expression, ↓ β-catenin, ↓ p-GSK3b, beneficial bacterial genera enrichment in the gut	[151]
	*L. casei* BL23	female C57BL/6 mice	antiproliferative and immunomodulatory effect, ↓ IL-22, ↑ caspase-7,-9, ↑ Bik, ↓ gut dysbiosis	[117]
	*L. casei* LH23	female C57BL/6J with DSS-induced cancer	↓ macrophages (CD11b^+^F4/80^+^) numbers, ↓ pro-inflammatory cytokines, ↓ MPO activity, ↑ Tregs, ↑ SCFAs, ↑ histone H3K9 acetylation in colon tissues	[271]
	*L. casei* subsp. *rhamnosus* (Lcr35)	CT26 tumor-bearing BALB/c mice	↓ diarrhea severity and intestinal mucositis after FOLFOX treatment, ↓ NF-κB pathway, ↓ TNF-α, ↓ IL-6, gut microbiota modulation	[224]
	*L. coryniformis* MXJ32	male C57BL/6 mice with AOM/DSS- induced cancer	↓ tumor number, intestinal barrier damage prevention, ↑ TJ proteins (occludin, claudin-1, and ZO-1) expression, ↓ inflammation, ↓ pro-inflammatory cytokines (TNF-α, IL-1β, IL-6, IL-γ, and IL-17a), ↓ chemokines, ↑ SCFAs-producing bacteria/↓ pro-inflammatory bacteria abundance	[243]
	*L. fermentum* ZS40	male C57BL/6 J mice with AOM/DSS- induced cancer	↓ colonic lesions, ↓ TNF-α, ↓ IL-1β, ↓ NF-κB pathway, ↓ COX-2 expression	[148]
	*L. gallinarum*	male and female Apc^Min/+^ C57B/6 mice	↓ tumor number and size, ILA enrichment in the gut	[237]
	*L. helveticus* NS8	C57BL/6 mice with AOM/DSS- induced cancer	↓ tumor number, ↑ apoptosis, ↓ NF-κB pathway, ↑ IL-10, ↓ IL-17-producing T cells, dysbiosis reversion	[120]
	*L. paracasei* R3	male MC-38 tumor-bearing C57BL/6 SPF grade mice	tumor-suppressive activity	[124]
	*L. plantarum* AS1	male albino Wistar with DMH-induced cancer	↓ lipid peroxidation, ↑ antioxidant enzymes (SOD, GST, catalase) and marker enzymes (ALP, ACP) activities, ↓ total number of tumors in AS1 pre- and post-treated rats in a time-dependent manner	[261]
	*L. plantarum* L168	C57BL/6 (B6) mice	↓ tumor growth, enhancement of CD8+ T cells function due to ILA secretion	[207]
	*L. plantarum* YYC-3	C57BL/6 APC^Min/+^ mice	mucosal damage prevention, dysbiosis restoration, ↓ NF-κB and Wnt pathways, ↓ pro-inflammatory cytokines (IL-6, IL-17, and IL-22), ↓ pro-inflammatory cells infiltration	[242]
	*L. plantarum* A, *L. rhamnosus* b	female CT26 tumor-bearing BALB/c mice	↓ tumor cell growth, prolonged survival time, ↑ CD8^+^ T and NK cell infiltration into TME, ↑ IFN-γ production, ↑ Th1-type CD4^+^ T differentiation	[275]
	*L. reuteri*	female BALB/c mice with TNBS-induced colitis	↓ intestinal inflammation mediated by Histamine H2 Receptor	[281]
	*L. rhamnosus* 231	male Wistar rats with MNNG-induced cancer	↓ fecal azoreductase and nitroreductase activity, ↓ GST, ↑ GSH, ↓ inflammation	[288]
	*L. rhamnosus* AFY06	C57BL/6 mice with AOM/DSS- induced cancer	↓ tumor incidence, ↓ pro-inflammatory cytokines, ↓ IkBb, p65, p50, p52, *Bcl-2*, and *Bcl-xL* expression, ↑ Bid and *CASP-8*	[279]
	*L. rhamnosus* GG	Sprague Dawley rats	↓ tumor incidence, ↑ apoptosis, ↓ NF-κB pathway	[114]
		C57BL/6 mice with AOM/DSS- induced cancer	↑ colonic CD8^+^ T-cell responses dependent on dendritic cell activation mediated via TLR-2	[272]
	*L. rhamnosus* LS8	C57BL/6 male mice with AOM/DSS- induced cancer	↓ tumor formation, goblet cell loss prevention, ↑ TJ proteins (ZO-1, occludin, and claudin-1) expression, dysbiosis reversion, ↓ inflammation, ↓ TLR4/NF-κB, ↓ pro-inflammatory cytokines, ↓ chemokines	[278]
	*L. rhamnosus* (Probio-M9)	CT26 tumor-bearing SPF BALB/c mice	enhanced immunotherapy response, ↑ beneficial microbes and metabolites in the gut, ↑ CTLs infiltration in the TME	[273]
		female C57BL/6NCrSlc mice with AOM/DSS- induced cancer	accelerated recovery of the gut microbiota composition and function	[241]
	*L. salivarius* Ren	male F344 rats	↓ cancer incidence, gut microbiota modulation	[232]
	*L. rhamnosus* MD14, *L. plantarum* GMD	male Sprague Dawley rats with DMH- induced cancer	↓ aberrant crypt foci, ↓ fecal pH, ↑ fecal LAB, altered fecal enzymes activities, gut microbiota modulation	[235]
	*Lactococcus lactis*	BALB/c mice with DMH-induced cancer	↑ CAT activity, ↓ H_2_O_2_ levels, ↓ colonic damage, ↓ inflammation, ↓ tumor incidence	[262]
	*Ligilactobacillus salivarius* LZZAY01	male C57BL/6J mice with AOM/DSS- induced cancer	↑ autophagy, ↑ apoptosis, ↑ intestinal TJs, ↓ intestinal barrier degradation, gut microbiota abundance modification, ↓ inflammatory reactions	[162]
	*Limosilactobacillus* *fermentum* GR-3	female C57BL/6J mice with AOM/DSS- induced cancer	↓ intestinal barrier disruption, ↓ tumor incidence, ↓ oxidative stress, ↓ inflammation, ↑ apoptosis, gut microbiota modulation, ↑ beneficial metabolites (SCFAs, ICA, IPA, vitamin B12 and vitamin D3), ↓ harmful secondary bile acids	[239]
	*Saccharomyces boulardii*	C57BL/6J Apc^Min/+^ mice	↓ intestinal tumor growth	[138]
		female C57BL6 mice with DSS-induced cancer	↓ histological damage, mucosal recovery restoration, ↓ VEGF-induced angiogenesis	[163]
		C57BL/6 mice with AOM/DSS- induced cancer	↓ carcinogenesis, ↓ TNF-α, ↓ IL-6, gut microbiota alterations	[236]
	*S. cerevisiae*	C57BL/6 mice with AOM-induced cancer and APC^Min/+^ mice	↓ carcinogenesis, ↑ apoptosis, ↓ NF-κB pathway, gut microbiota and intestinal immunity modulation	[139]
	*S. cerevisiae* SC-2201	male C57BL/6N mice with AOM/DSS- induced cancer	↓ colonic shortening and histological damage, ↓ pro-inflammatory mediators (IL-1β, IL-6, COX-2, VEGF, NBD, LRR, and NLRP3) expression, gut microbiota modulation	[166]
	*Streptococcus* *thermophilus*	male C57BL/6 mice with AOM-induced cancer and male Apc^Min/+^ mice	protective effect against intestinal tumorigenesis via β-galactosidase secretion, gut microbiota modulation, ↓ Hippo oncogenic pathway, OXPHO activation	[137]
	**Mixed formulations**			
	Mix I: *lactobacilli* and *bifidobacteria* Mix II: *bifidobacteria*	model 1: female C57BL/6 J mice with AOM/DSS- induced cancer model 2: female MC-38 tumor-bearing mice	**Mix I**: significant antitumor effects in the model 2, associated with microbiota-driven mechanisms **Mix II**: more effective in the model 1, ↓ colonic inflammation, tumor development prevention	[240]
	*Bifidobacteria* spp. cocktail	female BALB/c mice with AOM/DSS- induced cancer	colon length restoration, ↓ tumor incidence	[142]
	*B. bifidum* H3-R2 and *Lactococcus lactis* KLDS4.0325	male C57 BL/6 J mice with AOM/DSS- induced cancer	tissue damage relief, ↓ pro-inflammatory cytokines, ↑ anti-inflammatory cytokines, ↓ MPO activity, ↓ HIF-1α level, ↑ colonic TJ proteins, ↓ NLRP3 inflammasome, gut microbiota imbalance improvement	[144]
	GM-LAB	BALB/c mice with DMH-induced cancer	↓ intestinal damage, antioxidant enzyme activities modifications, ↑ anti-inflammatory cytokines	[258]
	*L. acidophilus* ATCC 314 and *L. fermentum* NCIMB 5221	male wild-type C57BL/6J– Apc^Min/+^ mice	↓ intestinal tumor multiplicity, ↓ proliferation markers (β-catenin and Ki-67)	[141]
	*L. rhamnosus* GG and *L. plantarum* AdF10	female Sprague Dawley rats with DMH-induced cancer	↑ antioxidant enzymes (GSH, GPx, GST, SOD, CAT) activities, ↑ p53-mediated apoptotic pathway	[154]
	Probiotic cocktail (*lactobacilli* and *bifidobacteria*)	Sprague Dawley rats with DMH-induced cancer	gut microbiota alteration, ↑ MUC2, ↑ ZO-1, ↑ occludin, ↑ TLR2, ↓ TLR4, ↓ COX-2, ↓ β-catenin	[233]
	Probiotic mixture VSL#3	male Sprague Dawley rats with TNBS- induced cancer	↓ intestinal damage, ↑ VDR expression, ↑ gut microbiota species richness and diversity, ↓ ALP levels, ↑ angiostatin expression in the colon	[231]
		male Wistar rats with DSS-induced cancer	↓ MPO activity, ↓ iNOS, ↓ COX-2, ↓ NF-κB pathway, ↓ TNF-α, ↓ IL-6, ↓ p-Akt, ↑ IL-10	[267]
		female C57BL/6 mice with DSS-induced cancer	↓ inflammation, ↓ colonic lesions, ↓ TNF-α, ↓ IL-6, ↓ IL-1β, ↓ COX-2, ↑ IL-10	[282]
	Probiótico 20 bi	male F344 rats with DMH-induced cancer	↓ aberrant crypt foci, ↓ tumor malignancy progression, 5-FU antitumor effect enhancement	[221]
		male C57BL/6 J mice	↓ NF-κB pathway, mitigation of mucin depletion, ↑ Ki-67 production	[249]
**Liver**	*L. plantarum* EMCC-1039	male Wister rats with TAA-induced cancer	↓ *TLR4*, *CXCL9* and *PREX-2* expression, liver function improvement	[266]
	*L. rhamnosus* ATCC 53103	male Swiss mice with induced HCC via DEN and CCl_4_ injection	gut leakage prevention, ↓ iNOS and IL-6 levels, ↓ intestinal and liver tissue inflammation	[285]
	Probiotic mix	male C57BL6/N mice	↓ tumor size and weight, ↓ IL-17, ↑ IL-10, ↓ pro-angiogenic genes expression, gut microbiota modulation, ↓ Th17 differentiation in the gut	[234]
	*Weizmannia coagulans* MZY531	female HT-22 tumor-bearing BALB/c mice	↓ tumor weight and size, ↓ pro-inflammatory cytokines, ↑ caspase-3, gut microbiota remodeling, ↑ AMPK/mTOR autophagy-dependent pathway, TLR4/MyD88/TRAF-6/NF-κB and JAK2/STAT3 inflammatory pathways regulation	[161]
**Pancreatic**	*L. rhamnosus* GG	female C57BL/6 Panc-02 tumor- bearing mice	↓ intratumor-promoting *Proteobacteria* and microbiota-derived LPSs, ↓ tumoral TLRs activation, ↓ PD-L1 and IL-1β expression by tumor cells, improved cytotoxic T lymphocytes infiltration in tumors	[274]
	**Mixed formulations**			
	*L. casei* ATCC 39392 and *L. reuteri* ATCC 23272	BxPC-3 tumor- bearing Balb/c mice	↓ TLR4 leading to gut microbial and metabolic homeostasis regulation	[265]
	*L. reuteri* GMNL-89 and *L. paracasei* GMNL-133	*P. gingivalis*-treated KC mice	↓ carcinogenesis	[213]
		KC transgenic mice	↓ PanIN formation following probiotics and gemcitabine combination, ↓ vimentin and Ki-67 expression, ↓ AST, ↓ ALT levels	[214]
	Probiotic blend	female BxPC-3 tumor-bearing Balb/c mice	↑ species richness and SCFAs producing-bacteria in fecal microbiota, ↑ phosphatidylcholine and phosphatidylethanolamine levels, ↓ amino acids (glutamic acid, aspartic acid, threonine and serine) levels	[245]

↑: induction, increase or up-regulation, ↓: inhibition, decrease or down-regulation.

## Abbreviations

The following abbreviations are used in this manuscript:

5-FU	5-Fluorouracil
5-HT	5-Hydroxytryptamine (Serotonin)
AAPH	2,2′-Azobis (2-Amidinopropane) Dihydrochloride
ACP	S-Acetyltransferase
ALP	Alkaline Phosphatase
AMPs	Antimicrobial Peptides
AOM	Azoxymethane
Apc	Adenomatous polyposis coli
ARE	Antioxidant Response Element
Bcl-2	B-cell Lymphoma-2
Bcl-xL	B-cell Lymphoma-xL
CA	Cholic Acid
CAC	Colitis-Associated Colorectal Cancer
CAT	Catalase
CEA	Carcinoembryonic Antigen
CFS	Cell-free Supernatant
cIAP2	Cellular Inhibitor of Apoptosis Protein 2
CLA	Conjugated Linoleic Acid
CNS	Central Nervous System
COX-2	Cyclooxygenase-2
CRC	Colorectal Cancer
CREB	cAMP Response Element-Binding Protein
CTLs	Cytotoxic T Lymphocytes
CXCR-4	C-X-C Chemokine Receptor type 4
DAMPs	Damage-Associated Molecular Patterns
DEPs	Differentially Expressed Proteins
DMH	1,2-Dimethylhydrazine
DSS	Dextran Sodium Sulfate
EcN	*Escherichia coli* Nissle 1917
EFSA	European Food Safety Authority
EGFR	Epidermal Growth Factor Receptor
EMT	Epithelial–Mesenchymal Transition
ENS	Enteric Nervous System
EPS	Exopolysaccharides
ETEC	Enterotoxin-producing *Escherichia coli*
EVs	Extracellular Vesicles
FAO	Food and Agriculture Organization
GABA	Gamma-Aminobutyric Acid
GABA_A_R	Ionotropic GABA_A_ Receptor
GABA_B_R	Metabotropic GABA_B_ Receptor
Gal	Galunisertib
GBA	Gut-Brain Axis
GI	Gastrointestinal
GIT	Gastrointestinal Tract
GPR	G-Protein Coupled Receptor
GSH-Px	Glutathione Peroxidase
GST	Glutathione S-Transferase
HER2	Human Epidermal Growth Factor Receptor 2
IBD	Inflammatory Bowel Disease
ICA	Indole-3-Carboxylic acid
ICD	Immunogenic Cell Death
IECs	Intestinal Epithelial Cells
IFN-γ	Interferon-γ
IGF-1	Insulin-like Growth Factor-1
IL	Interleukin
ILA	Indole-3-Lactic Acid
IM	Intestinal Mucositis
iNOS	inducible Nitric Oxide Synthase
IPA	Indole-3-Propionic Acid
ISAPP	International Scientific Association of Probiotics and Prebiotics
Keap1	Kelchlike ECH-associated protein-1
LAB	Lactic Acid Bacteria
LDH	Lactate Dehydrogenase
LPS	Lipopolysaccharide
MAPK	Mitogen-Activated Protein Kinase
MDA	Malondialdehyde
METTL3	Methyltransferase-like 3
MHC	Major Histocompatibility Complex
MMP	Mitochondrial Membrane Potential
MNNG	N-Methyl-N’-nitro-N-nitrosoguanidine
MPTP	Mitochondrial Permeability Transition Pore
mTOR	Mammalian Target of Rapamycin
MyD88	Myeloid Differentiation Factor 88
NF-κB	Nuclear Factor-kappa B
NFKB1	Nuclear Factor-kappa B subunit 1
NK cells	Natural Killer cells
NLRP3	NOD-like Receptor Protein 3
NOD2	Nucleotide-binding Oligomerization Domain-containing protein 2
Nrf-2	Nuclear Factor Erythroid 2-related factor 2
ODC	Ornithine Decarboxylase
PD-L1	Programmed Death-Ligand 1
PI3K	Phosphatidylinositol 3-Kinase
PINK1	PTEN Induced Kinase 1
PKB	Protein Kinase B (Akt)
PTEN	Phosphatase and Tensin Homolog
PTGS2	Prostaglandin-Endoperoxide Synthase 2
ROS	Reactive Oxygen Species
RXR	Retinoid X Receptor
SCFAs	Short-Chain Fatty Acids
SOD	Superoxide Dismutase
SPF	Specific Pathogen-Free
TAA	Thioacetamide
TCA	Tricarboxylic Acid Cycle
TGF-β	Transforming Growth Factor-β
Th1	Type 1 T helper cells
Th17	Type 17 T helper cells
TJ	Tight Junction
TLR	Toll-like Receptor
TME	Tumor Microenvironment
TNBS	Trinitrobenzene Sulfonic Acid
TNF-α	Tumor Necrosis Factor-α
TRAIL	TNF-Related Apoptosis-Inducing Ligand
Treg	T regulatory cells
uPA	Urokinase Plasminogen Activator
uPAR	Urokinase Plasminogen Activator Receptor
VEGFR	Vascular Endothelial Growth Factor Receptor
VM	Vasculogenic mimicry
WHO	World Health Organization
